# New Hydrogels and Formulations Based on Piperonyl-Imino-Chitosan Derivatives

**DOI:** 10.3390/polym15030753

**Published:** 2023-02-01

**Authors:** Daniela Ailincai, Irina Rosca

**Affiliations:** “Petru Poni” Institute of Macromolecular Chemistry, 700487 Iasi, Romania

**Keywords:** hydrogels, antifungal, biodegradable, piperonal, Amphotericin B

## Abstract

*Candida* infections have been always a serious healcare related problem. The present study reports the preparation of hydrogels and formulations based on piperonyl-imino-chitosan derivatives and Amphotericin B drug for the treatment of *Candida* infections. The hydrogels were obtained by the imination reaction of chitosan with piperonal monoaldehyde, followed by the self-assembling of the resulted imines, while the formulations were obtained by an in situ hydrogelation method of chitosan with piperonal in the presence of Amphotericin B antifungal drug. The structural characterization of both hydrogels and formulations by Fourier transform infrared spectroscopy and Nuclear magnetic resonance spectroscopy revealed the formation of imine units between the reagents, while their supramolecular characterization using polarized optical microscopy and wide angle X-ray diffraction demonstrated that hydrophilic/hydrophobic segregation is the process which governed the formation of gel like systems. The systems were further investigated from the point of view of their further applications revealing that they were biodegradable, presented high swelling ability and were able to release the antifungal drug in a sustained manner, presenting promising antifungal activity against five *Candida* strains.

## 1. Introduction

Candida species are opportunistic pathogens which represent a major cause of morbidity worldwide being considered a serious problem related to human healthcare [1]. Candida pathogens cause diverse problems such as oral candidiasis, vaginitis, skin candidiasis and last but not least candidimia (which represents infection of the blood stream) [2]. There are numerous Candida species, among which *Candida albicans*, *Candida glabrata* and *Candida parapsilosis* are the most frequently encountered [3]. 

Candida infections were always a serious problem. Although, in the last decade their incidence increased a lot, being favored by the increase of the number of immunocompromised patients, those who require organs transplantation or artificial feeding methods through catheters [4]. Even if there are a multitude of antifungal drugs such as fluconazole, Amphotericin B, Flucytosine and so on, there is a continuous need for the development of new formulations containing these drugs in order to reduce their side effects, to protect them on their way to the active site and last but not least to reduce the frequency of their administration [5]. 

One of the most effective way to achieve that, is by using hydrogels as matrix for the encapsulation of antifungal agents. Hydrogels are materials used at large scale for a wide range of applications, such as actuators [6], waste water treatment [7], long-lasting antimicrobial materials [8] and drug delivery systems [9], due to their versatile properties. Among the polymers used for hydrogels’ preparation, chitosan (C), which is a polysaccharide derived from chitin [10], is intensely used due to its intrinsic properties: biocompatibility, biodegradability and very important similarity with the extracellular matrix from compositionally and mechanical properties points of view [11,12,13]. Previous studies of our group revealed that chitosan or oligochitosan based hydrogels present high potential of being used as materials with biomedical applications per se [14] or acting as matrix for the encapsulation of different drugs [15,16,17]. The systems presented the ability to release the encapsulated drugs in a sustained manner, due to the interactions developed between the matrix and the drug. 

In this context, the present study aimed to synthesize new hydrogels based on chitosan and piperonal (P) monoaldehyde which can be successfully used as excipients for the encapsulation of Amphotericin B (AmB) antifungal drug, meeting the requirements for such materials, such as biocompatibility, biodegradability, to be inert and not to interfere with the drug in a manner in which would affect its biological activity [18,19,20]. 

The synthesized systems are innovative, no hydrogels based on C and P and no formulations based on C, P and Am B drug, being reported up to now. Moreover, the obtained materials had a rational design which took into consideration the fulfillment of the prerequisites necessary for the development of formulations able to release the antifungal agent in a sustained manner.

The use of P, which is a natural origin monoaldehyde, frequently used in fragrances and flavors, brings the advantage of biocompatibility, the reported value of LD50 for P being of 2700 mg/kg [21]. In the designed systems, P moieties are linked on chitosan backbone through reversible imine linkages which should assure the degradation of the matrix in the biological environment which is mostly made of water [18]. Moreover, the choice of the drug was not randomly done. The AmB is a polyene type antifungal drug, containing ten hydroxyl groups in its structure, which may interact with the hydroxyl, amine and amide groups of chitosan, creating a new network of hydrogen bonds, besides the one of the biopolymer [20]. Furthermore, AmB has a carboxylic group, being an anionic drug, which should facilitate the formation of electrostatic interactions with the polycationic chain of chitosan [20]. These data create the premises of the obtaining of systems able to release the antifungal drug in a sustained manner, fulfilling the requirements of biodegradability and biocompatibility imposed to materials for biomedical applications. 

## 2. Materials and Methods

### 2.1. Materials

Chitosan (297 kDa, DD = 88%), piperonal, ethanol, Amphotericin B (97%), lysozyme from chicken white egg were purchased from Sigma Aldrich and used as received. 

### 2.2. The Synthesis of the Reference Hydrogels

**Scheme 1:**
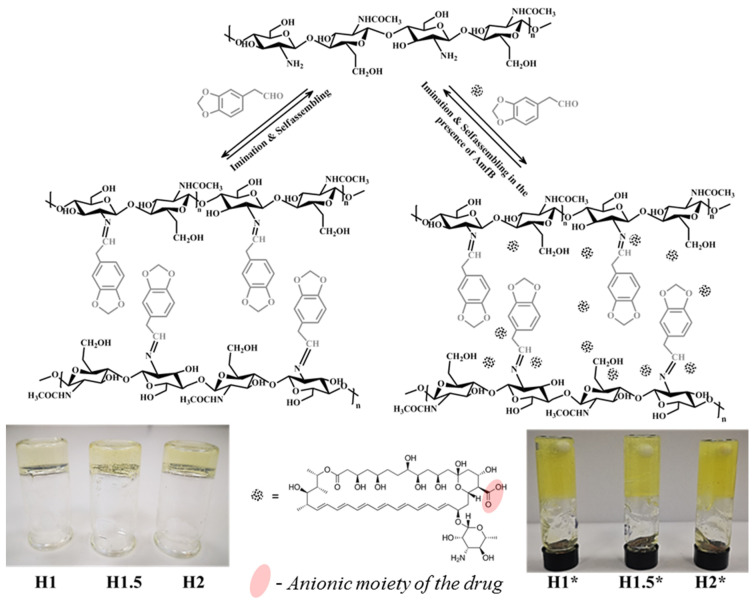
The synthesis of the piperonyl-iminochitosan derivatives and the formation of the hydrogels and the corresponding formulations.

A series of three hydrogels were synthesized by the imination reaction between C and P monoaldehyde, followed by the self-assembling of the formed imine units. The hydrogels components were mixed in different molar ratios between their functionalities, according to Table 1. As an example, the experimental procedure for the **H1** hydrogel was the following: 120 mg C was dissolved in 4 mL acetic acid aqueous solution 0.7%, on a magnetic stirring plate. After chitosan’s solubilization, the temperature was raised up at 65 °C, when 88 mg P solution in 4.4 mL ethanol was added to the reaction medium. A hydrogel was formed for the sample **H1** after 15 min, while for the **H1.5** and **H2** samples, after ~1 h. The hydrogels passed the inverted tube tests, forming homogenous and clear hydrogels. Pictures of the obtained hydrogels are presented in Scheme 1. 

### 2.3. The Synthesis of the AmB Formulations

The experimental procedure followed for the reference hydrogels was repeated in the presence of AmB antifungal drug, with slight modifications meant to maintain the drug soluble (Table 1). Therefore, for the H1* the procedure was as follows: 120 mg C were dissolved in 4 mL acetic acid aqueous solution 0.7%, and the solution temperature was raised to 65 °C. To the C solution, 4 mg AmB dissolved in 40 µL DMSO was added, under strong magnetic stirring. After ~5 min, to this mixture the corresponding amount of aldehyde (88 mg) dissolved in 4.4 mL ethanol was added. A hydrogel was formed after 30 min for H1*, while for H1.5* and H2*, the hydrogels formed in 3 and respectively 6 h (Scheme 1).

### 2.4. Methods

In order to obtain the hydrogels in dried state, further called xerogels, the samples were freeze-dried using a LABCONCO FreeZone FreezeDry System (Canada), at −54 °C and 1.510 mbar, for 24 h. The FTIR spectra of the starting materials (C, P, AmB), of the reference xerogels (**H1**, **H1.5** and **H2**) and formulations (H1*, H1.5* and H2*), were recorded on a FTIR Bruker Vertex 70 Spectrophotometer (Ettligen, Germany), with a ZnSe single reflection ATR accessory. The NMR spectra were recorded on a Bruker Avance DRX 400 MHz Spectrometer (Bruker, Germany) equipped with a 5 mm QNP direct detection probe and z-gradients on samples prepared directly in deuterium oxide. The supramolecular architecture of the synthesized systems, hydrogels and formulations, was evaluated by polarized optical microscopy on a Leica DM2500 microscope, (Hamburg, Germany) and by X-ray diffraction on a Rigaku Miniflex 600 diffractometer using CuKα-emission in the range 2–40° (2θ) with a scanning step of 0.0025° and a recording rate of 1°/min. The morphology was evaluated using a Scanning Electron Microscope SEM EDAX—Quanta 200 (Eindhoven, Germany) on xerogels. Atomic force microscopy (AFM) was performed using a Ntegra Spectra Instrument (NT-MDT, Russia) in the semi-contact mode, at room temperature and using a silicon cantilever (NSG 10) on hydrogel samples deposited on mica.

The swelling behaviour of the reference hydrogels was evaluated in PBS, pH = 7.4 by weighting from time to time the samples immersed into phosphate buffer saline solution (PBS), in order to calculate the mass equilibrium swelling according to the following equation:

(1) $$MES=\frac{W_{t\;}-W_{0}}{W_{0}}\cdot 100$$

where *MES* = mass equilibrium swelling the hydrogel; *W_t_* = the weight of the hydrogels at a certain time and *W*_0_ = the initial weight of the hydrogel.

The in vitro enzymatic degradation of the reference hydrogels was monitored by evaluating the mass loss of pieces of hydrogels immersed and kept for different time intervals in lysozyme buffer solution. For comparison, one sample was also evaluated from the point of view of hydrolytic degradation. Pieces of hydrogels containing an estimated dry mass of 8 to 10 mg each were added in 8 to 10 mL of lysozyme solution (376 U/L) and kept at 37 °C for several days (1, 3, 6, 9, 21 days). At every 3 days, the lysozyme solution was replaced with a freshly prepared one, in order to avoid the decrease of enzyme activity. At certain time intervals the samples were removed, washed with double distilled water to remove the salts from the buffer and submitted to gravimetric measurements and SEM analysis. The mass loss was calculated using the following equation:

(2) $$W_{loss}=\frac{W_{0\;}-W_{t}}{W_{0}}\cdot 100$$

where *W_loss_* = the weight loss of the hydrogel; *W*_0_ = the initial weight of the hydrogel; *W_t_* = the weight of the hydrogels at a predetermined moment. 

The in vitro release kinetics of the AmB drug from the formulations was monitored in PBS (pH = 7.4), at 37 °C. The release kinetics curve was built using a previously drawn calibration curve based on the adsorption maximum of AmB from 408 nm [17]. Pieces of hydrogels containing a known amount of AmB were immersed into 10 mL PBS and kept at 37 °C. From time to time, 2 mL of supernatant was removed and replaced with fresh PBS, in order to maintain the constant volume at 10 mL [17]. The concentration of the drug in the supernatant was determined by UV-Vis spectroscopy, according to Lambert–Beer law, using the absorbance value of the maximum from 408 nm. 

The drug release kinetic data were analyzed by fitting the experimental data on the following mathematical models: Higuchi, Korsmeyer-Peppas, Zero order and Hixson-Crowell:

(i) Zero order model: $Q_{t}=K_{0}\cdot t$, where *Q_t_* is the amount of drug dissolved in the time *t* and *K*_0_ is the zero order release constant.
(ii) Higuchi model: $Q_{t}=K_{H}\cdot t^{\frac{1}{2}}$, where *Q_t_* is the amount of drug released in the time *t* and *K_H_* is the Higuchi dissolution constant.
(iii) Hixson-Crowell model: $W_{0}^{1/3}-W_{t}^{1/3}=K\cdot t$, where *W*_0_ is the initial amount of drug in the formulation, *W_t_* is the remaining amount of drug in the formulation at time *t* and *K* is a constant.
(iv) Korsmeyer-Peppas model: $\frac{M_{t}}{M_{\infty }}=K\cdot t^{n}$, where *M_t_*/*M*_∞_ is the fraction of drug released at the time *t*, *K* is the release rate constant and *n* is the release exponent.

The antimicrobial activity screening of the samples was determined by disk diffusion assay [20,21,22] against five representative fungal strains: *Candida albicans* ATCC10231 (*C. albicans* ATCC10231), *Candida albicans* ATCC90028 (*C. albicans* ATCC90028), *Candida glabrata* ATCC20019 (*C. glabrata* ATCC20019), *Candida glabrata* ATCC15126 (*C. glabrata* ATCC15126), and *Candida parapsilosis* ATCC2201 (*C. parapsilosis* ATCC2201). All microorganisms were stored at −80 °C in 20% glycerol. The yeast strains were refreshed on Sabouraud dextrose agar (SDA) at 37 °C. Microbial suspensions were prepared with these cultures in sterile solution to obtain turbidity optically comparable to that of 0.5 McFarland standards. Volumes of 0.1 mL from each inoculum were spread onto SDA plates and then the sterilized paper disks (6 mm) with an aliquot (50 μL) of the samples were added. To evaluate the antimicrobial properties, the growth inhibition was measured under standard conditions after 24 h of incubation at 37 °C. All tests were carried out in triplicate to verify the results. After incubation, the samples were analysed with SCAN1200^®^, version 8.6.10.0 (Interscience) and were expressed as the mean ± standard deviation (SD) performed with XLSTAT Ecology version 2019.4.1 software [22].

## 3. Results and Discussions

A series of three hydrogels (**H1**, **H1.5** and **H2**) and three formulations (H1*, H1.5* and H2*) were synthesized by the imination reaction between C and P monoaldehyde, followed by the self-assembling of the formed imine units in the absence or presence of AmB antifungal drug (Table 1). The systems were based on a rational design strongly related to the structural particularities of the components—the polycationic chain of chitosan and the anionic character of AmB which should facilitate drug-matrix interactions, and also to the structural similarities between the components: both chitosan and AmB presenting multiple hydroxyl groups which should facilitate the formation of hydrogen bonds between the drug and the polymeric matrix [15,16]. The hydrogelation consisted in the obtaining of the piperonyl-iminochitosan derivatives, followed by their selfassembling in the presence or absence of the AmB drug (Scheme 1). The hydrogelation occurred faster in the case of the reference hydrogels, compared to the DDSs, very probably because of the presence of Amphotericin B which is a drug with a large molecule and may hamper the selfassembling of the piperonyl-iminochitosan derivatives, leading to longer hydrogelation times. This being said, it can be concluded that through this rational design are fulfilled some mandatory requirements for the development of sustained release drug delivery systems. 

### 3.1. Structural Characterization by Fourier Transform Infrared Spectroscopy (FTIR) & Nuclear Magnetic Resonance Spectroscopy (NMR)

**Figure 1:**
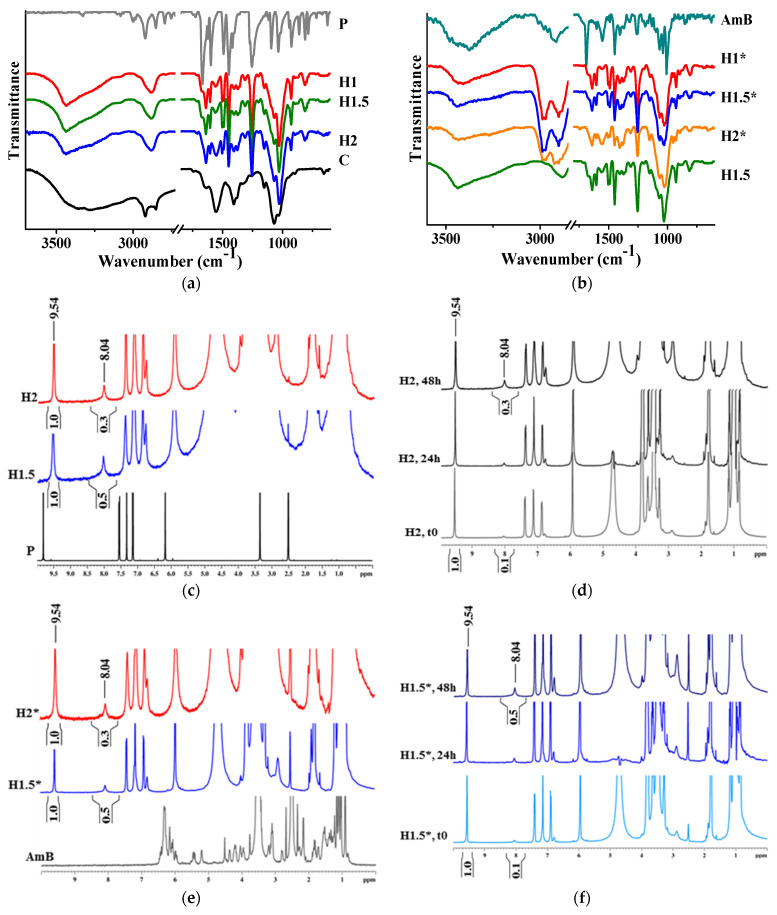
(**a**,**b**) FTIR & (**c**–**f**) NMR spectra of representative hydrogels and formulations.

The structural characterization of the xerogels was done in comparison with the starting materials: C and P monoaldehyde. As it could be observed, the P FTIR spectrum presented characteristic absorption bands according to its structure: at 1675 cm^−1^ a sharp band characteristic to the stretching vibration of the carbonyl group, while the bands from 1581 and 1496 correspond to the stretching of the aromatic C=C bonds [23,24]. The C spectrum presented an absorption band of low intensity at 1641 cm^−1^ characteristic to the stretching of the amide groups and a broad band in the 3700–2700 cm^−1^ domain with two maxima at 3351 and 3277 cm^−1^ due to the vibrations of the OH and NH2 groups, involved in hydrogen bonds (Figure 1a) [25,26]. Comparing the spectra of the xerogels with the ones of C and P, some modifications were observed, indicating the forming of imine linkages between the reagents, by the appearing of a new absorption band at 1638 cm^−1^ [27]. Moreover, some modifications in the hydrogen bonds environment, consequence of the imination, were also observed, by the changing in terms of peak shape and position in the 3700–2700 cm^−1^ spectral regions. Therefore, as already discussed, C presented two maxima in this spectral region, while the xerogels presented a broad band with one sharp maximum, at 3447 cm^−1^. This data indicated some morphological changes in the chitosan due to the grafting of piperonyl moieties on its chains [28]. Regarding the FTIR spectra of the formulations, similar changes were observed as in the case of the reference hydrogels, indicating the formation of the imine linkages even in the presence of AmB drug (Figure 1b).

For a deeper insight regarding the structure of the synthesized hydrogels, two hydrogels (**H1.5** and **H2**) were prepared in deuterium oxide and the NMR spectra were recorded. The spectra of the hydrogels presented the chemical shift corresponding to the imine proton at 8.04 ppm [11,19]. Due to the reversible character of the imine linkages, the chemical shift corresponding to the aldehyde proton was also presented into the spectra, indicating the formation of a dynamic system. The monitoring of the system, for several hours, revealed that the imination reaction is a process which evolves in time, reaching a maximum in 48 h (Figure 1d), the ratio between the integral of the imine proton to the one corresponding to the aldehyde increasing from 0.1 to 0.3 in the case of the **H2** hydrogel and from 0.1 to 0.5 in the case of the **H1.5** hydrogel (Figure 1d). The same behavior was observed also in the case of the formulations, the equilibrium of the imination reaction not being influenced by the presence of the AmB drug (Figure 1e,f). 

### 3.2. Supramolecular Architecture by Polarized Optical Microscopy (POM) and Wide Angle X-ray Diffraction (WXRD)

**Figure 2:**
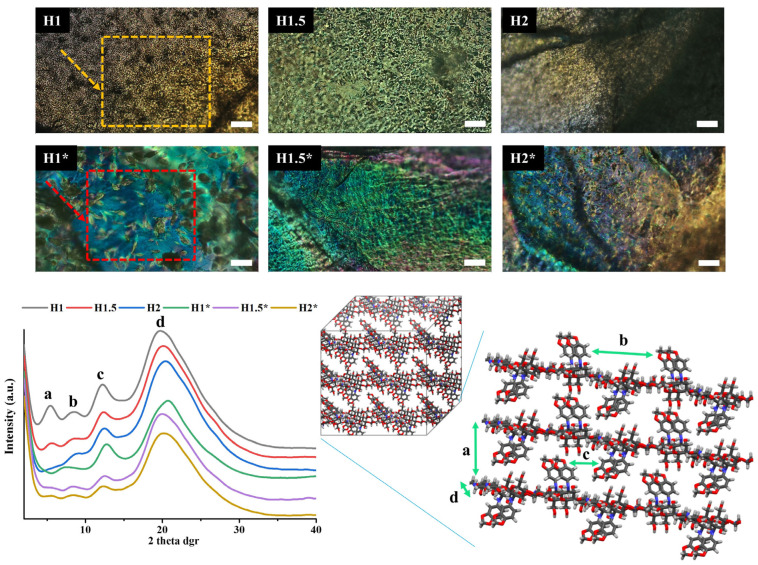
Diffractograms of the reference xerogels and formulations and POM images for the reference hydrogels and the formulations (scale bar 50 µm) (a, b, c, d represent the diffraction peaks and the corresponding *d*-spacings from the Bragg’s law). The regions indicated with arrows indicate strong birefringence.

POM was used in order to evaluate the supramolecular arrangement of the reference hydrogels and the formulations. The pure drug was also investigated from this point of view. The images indicated the crystalline nature of the AmB drug, by the presence of birefringence in polarized light, according to the literature [29]. The reference hydrogels also presented weak birefringence due to their ordered structure at supramolecular level [11,12,14]. On the other side, the formulations presented strong birefringence indicating a more ordered architecture, possible due to the presence of AmB which is highly crystalline (Figure 2). 

In order to evaluate the physical state of the encapsulated AmB drug into the formulations, the samples were submitted to wide angle X-ray diffraction analysis. The method was also used for the characterization of the reference xerogels, with the aim to understand their supramolecular ordering, observed by POM. 

The reference xerogels presented four diffraction peaks suggesting a supramolecular layered architecture, given by the segregation of the hydrophilic chitosan chains and hydrophobic imine units [30]. The diffraction peaks correspond to the following d-spacings: 16.4; 10.4; 7.2 and 4.5 Å respectively, attributed based on a HyperChem simulation to intra- and inter-molecular distances from the xerogels supramolecular architecture, according to the schematic representation from Figure 2. It was estimated that the imine units aggregated into ordered cluster, playing the role of crosslinking nodes. 

Regarding the formulations X-ray data, the first observation was that there are no diffraction peaks characteristic for the drug crystals, indicating once more that AmB was intimately encapsulated into the hydrogels matrix. However, considering that the formulations presented a more vivid birefringence in POM and FTIR spectra indicated reorganization of the H-bonds network, it can be envisaged that AmB bonded via H-bonds to the chitosan chains increasing the fraction of the ordered domains, but the presence of sub-submicrometric AmB crystals whose diffraction peaks were hidden under the high intensity peaks of the chitosan based matrix can’t be excluded. 

### 3.3. Morphological Study at Micro Level and Nano Level 

**Figure 3:**
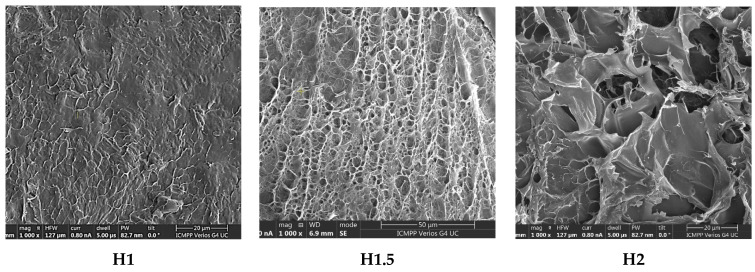

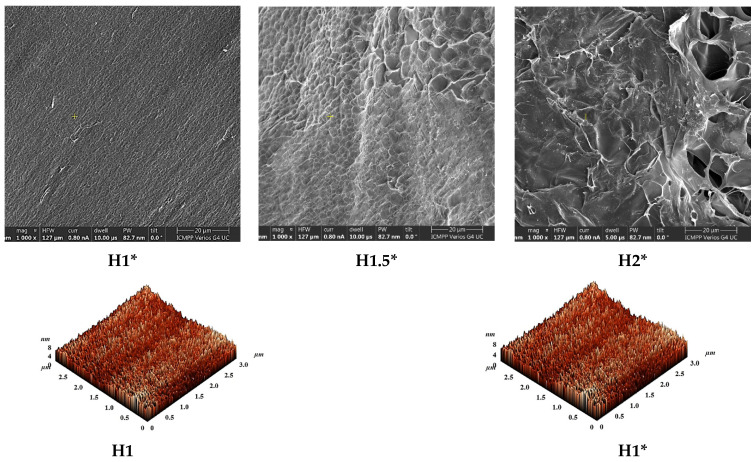
SEM microphotographs of the reference and formulations xerogels and AFM of two representative samples.

The morphological characterization of the hydrogels and their formulations was done at micro scale by scanning electron microscopy (SEM) on the corresponding xerogels. All the samples presented a porous morphology with micrometric pores. SEM allowed also the evaluation of the state of the encapsulated drug into the hydrogels. As it could be observed, no micrometric drug crystals were observed into the hydrogels, confirming that the AmB drug was intimately encapsulated at sub-micrometric level into the formulations (Figure 3). 

Further, the morphology of both formulations and the reference hydrogels was more deeply evaluated, at nanometric level, by atomic force microscopy (AFM) in topographic mode. The samples presented a granular morphology regardless the presence or absence of AmB drug. Representative images are presented in Figure 3 showing that the grains are uniformly distributed on the entire hydrogel’s surface. 

### 3.4. Swelling Behavior of the Reference Hydrogels

**Figure 4:**
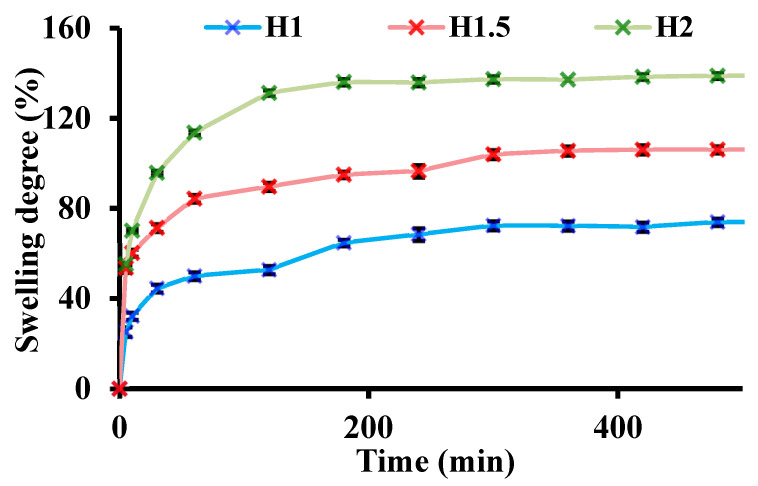
The swelling kinetics of the reference hydrogels in PBS.

To have an insight regarding the behavior of the hydrogels in the physiological medium, their swelling behavior was monitored in PBS solution of physiological pH. The obtained data revealed that the samples swelled with different ratios depending on the density of the piperonyl units on chitosan chain (Figure 4). Therefore, the **H1** sample reached a maximum swelling degree of 106%, while the sample **H2** led to a maximum value of 140% (Figure 4). All the samples, regardless the piperonal content, reached the maximum swelling degree in ~300 min. 

### 3.5. Enzymatic Degradation Study

**Figure 5:**
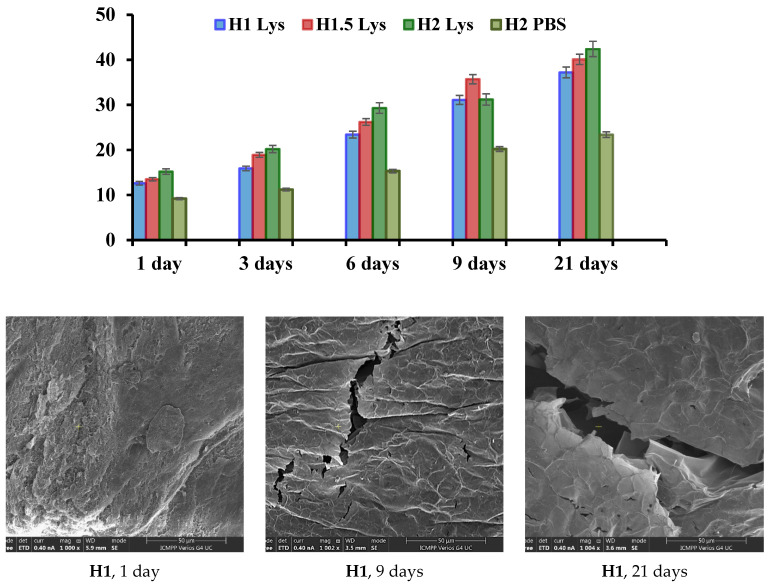

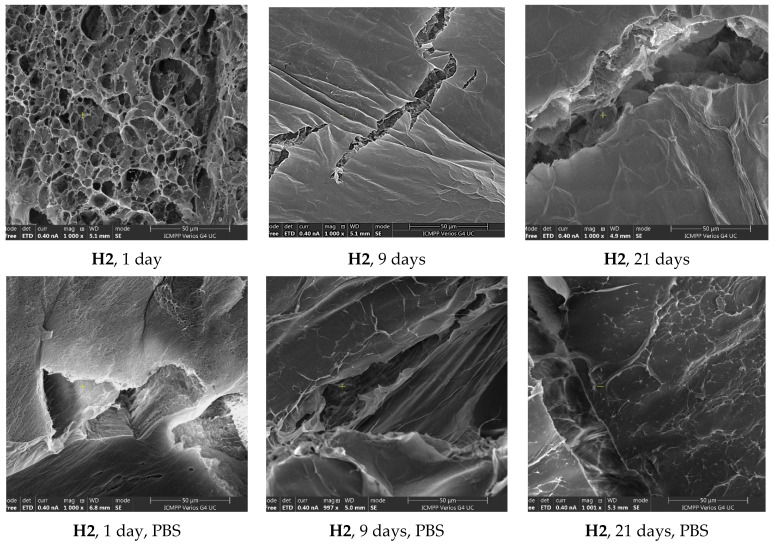
Mass loss of the samples during the in vitro enzymatic degradation tests and representative SEM images postdegradation.

The absence of matrix accumulation into the body is an important parameter which must be taken into consideration in the development of new drug delivery systems [31]. Therefore, the samples were evaluated from the point of view of their hydrolytic and enzymatic degradability. The experiments were done gravimetrically by determining the mass loss of the samples and from the morphological point of view by SEM. The results showed a positive influence of the enzyme on the degradability rate and extent, the presence of lysozyme leading to higher values of the mass loss, with a maximum of 42% for the **H2** sample compared to 23.4% in its absence (Figure 5). This can be explained by the larger pores of this sample and also the looseness of the network in comparison with the other samples, which allowed an easier diffusion of the enzime. Therefore, the hydrogels proved to be highly and rapidly biodegradable in the presence of lysozyme, making them suitable from bioapplications. 

The morphological changes of the hydrogels post hydrolytic or enzymatic degradation revealed the presence of cracks in the hydrogels’ structure, with the observation that the cracks were bigger in the case of the samples which were kept longer time in the presence of enzyme solution. 

### 3.6. In Vitro Drug Release

The AmB release ability of the designed formulations was evaluated by UV-VIS spectroscopy. The samples were able to release the encapsulated drug with different rates, depending mainly on their stiffness and hydrophilicity. All the samples followed the same release pattern, with a burst effect in the first 8 h, followed by a sustained release up to 10 days. 

**Figure 6:**
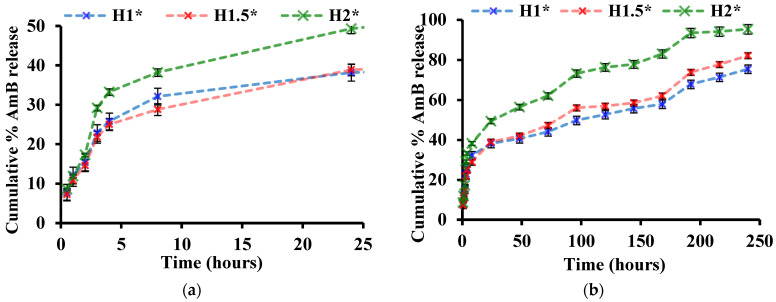
The experimental release kinetic curves for the formulations for the first 8 h (**a**) and for the 240 h during the experiment (**b**).

The sample H1*, which is characterized by the most numerous hydrophobic imine clusters which act as crosslinking nodes, released the encapsulated drug with the slowest rate, in 24 h releasing 38% from the total encapsulated amount. On contrary, the sample H2* released the drug faster, due to the looser network, which allowed the sample swelling and therefore drug’s dissolution, in 24 h releasing 49% from the total AmB amount (Figure 6). 

Taking all this into consideration, it can be concluded that the systems are versatile from the point of view of drug release rate, this parameter being governed by the intrinsic hydrophilicity of the matrix. 

### 3.7. The Investigation of the AmB Release Mechanism from the Formulations

A release of a drug from a polymeric matrix is a complex process which is governed by many factors regarding both the drug and the matrix characteristics. Therefore, in order to elucidate the most important factors which influence the release of AmB from the hydrogels, the kinetic data obtained experimentally by UV-VIS were fitted on several mathematical models such as: zero order, Korsmeyer-Peppas, Higuchi and Hixson Crowell models (Table 2). The experimental data were fitted on two different stages: 0–8 h and 24–144 h, respectively. 

The Higuchi model gives information regarding the influence of the diffusion process on the drug’s release from the matrix [32]. Therefore, analyzing the correlation coefficient for the samples on both stages, satisfactory values were obtained, in all cases the values of R^2^ being higher than 0.92. This data clearly indicates that on the whole release process of AmB drug, its diffusion from the matrix plays a crucial role. Further, the kinetic data were fitted on Korsmeyer Peppas model, in order to elucidate what type of diffusion is involved in the process [32,33,34]. The kinetic data fitted very well on the Korsmeyer-Peppas model, the values of the correlation coefficient being higher than 0.95 in the first stage and higher than 0.93 in the second one. A difference was observed in terms of n_r_ parameter which is higher than 0.5 in the first stage and lower than 0.45 in the second one. This indicates that in the first 8 h there are two major factors which govern the AmB release process: matrix swelling and drug’s diffusion, while in the second stage the drug release from the matrix follows a quasi Fickian diffusion mechanism.

The zero-order model led to high values of the correlation coefficient only in the second stage, fact which indicates that after 8 h there is a constant release of AmB as a function of time, influenced by the drug’s rate of solubilization [35]. 

The low values of the correlation coefficient obtained on the Hixson-Crowell model for the first stage, confirmed that the release is dictated in the first hours by drug’ solubilization and diffusion from the matrix [32,33,35]. In the second stage, after 24 h, the linear fitting of the kinetic data on the Hixson-Crowell model led to high values of the correlation coefficient, the R^2^ being higher than 0.94, revealing that in the second stage, the matrix starts to erode, influencing in a significant manner the drug release process. 

### 3.8. Antimicrobial Activity by Kirby Bauer Method

**Figure 7:**
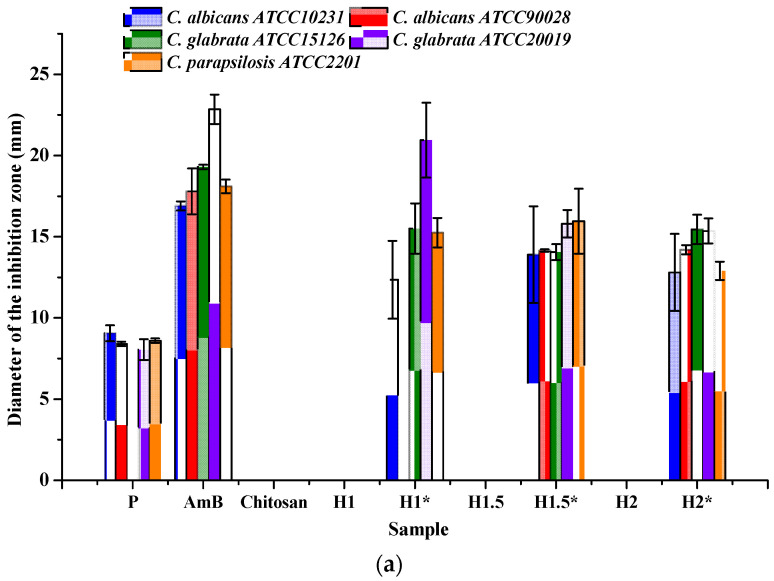

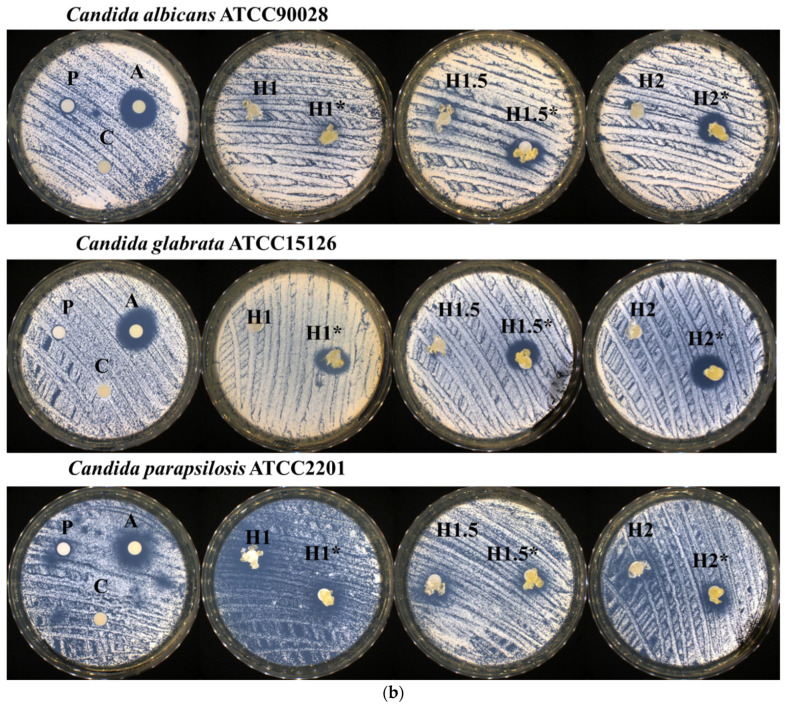
Antifungal activity (mm) of the samples against the reference strains (**a**) and representative pictures taken during the exeriments (**b**).

All the data proved that between the drug and the matrix there are strong interactions. Therefore, in order to evaluate if these interactions influence in a negative manner the antifungal activity of the drug, the hydrogels were evaluated on five *Candida* strains and the diameter of the inhibition zone was measured. The tests were done on hydrogels, formulations and their components of similar concentration as they are into the hydrogels. The obtained data are presented in Figure 7. Even if C was reported in the literature as presenting antifungal and antibacterial properties, this fact was not confirmed by this study, very probably because of differences between chitosan batches such as molecular weight and deacetylation degree, these two parameters being the ones which mostly influence its properties. AmB presented the standard antifungal activity for the tested concentration [36] and was efficient against all the tested yeast strains, being known that its mechanism of action consists in the fact that its molecule binds to the ergosterol in the fungal cell membrane, forming an aggregate that opens a transmembrane channel, causing the cytoplasmic contents to flow out and ultimately causing cell death. Piperonal nitro derivatives are also known to have antifungal activity against *Candida* species [37]. In this case, the tested concentration of P monoaldehyde had a small antifungal activity against 4 of the tested strains. 

The reference hydrogels didn’t present any antifungal activity, while the formulations were quite efficient against the tested strains, the most promising results being obtained in case of *C. glabrata* ATCC20019 (up to 23 mm of inhibition zone for H1* sample). The activity of the other two samples was similar one to another, revealing the fact that at 24 h during the experiment there is no significant difference in the amount of AmB released from the two different hydrogels. 

## 4. Conclusions

The study aimed to synthesize new hydrogels based on piperonyl-imino-chitosan derivatives, which were further used as excipients for the development of formulations with Amphotericin B antifungal drug. The systems had a rational design based on the structural similarities between chitosan and Amphotericin B which should allow the formation of hydrogen bonds leading to the obtaining of sustained release drug delivery systems. The obtained data regarding the morphological and supramolecular characterization of the formulations revealed the uniform fine distribution of the drug into the matrix, as demonstrated by SEM, POM and WXRD. Moreover, the evaluation of the swelling ability, biodegradability and in vitro release tests demonstrated that these systems are adequate for bioapplications, being able to release the antifungal Amphotericin B drug in a sustained manner. Moreover, the evaluation of the antifungal activity of the formulations led to quite high values of the inhibition zones against five *Candida* strains revealing that drug-matrix interactions lead to a prolonged release of the antifungal drug into the medium. 

## Figures and Tables

**Table 1:** The composition of the reference hydrogels and formulations.

Sample Code	m_C_ (mg)	V_water_ (mL)	V_acetic acid_ (μL)	m_P_ (mg)	V_ethanol_ (mL)	m_AmB_ (mg)	V_DMSO_ (μL)	m_1_^1^ (mg)	m_2_^2^ (mg)	η (%)
**H1**	120	4	28	88	4.4	-	-	208	207.5	99.75
**H1.5**	120	4	28	66	3.3	-	-	186	186	100
**H2**	120	4	28	44	2.2	-	-	164	163.7	99.91
H1*	120	4	28	88	4.4	4	40	212	211.8	99.9
H1.5*	120	4	28	66	3.3	4	40	190	189.5	99.73
H2*	120	4	28	44	2.2	4	40	168	167.8	99.88

^1^**m_1_**—the amount of solid substances added to the reaction; ^2^**m_2_** = the xerogel mass after freeze-drying.

**Table 2:** The data resulted by fitting the experimental kinetic data for AmB on various mathematical models for the two stages (0–8 h) and (24–144 h).

	Code	Zero Order		Higuchi Model		Hixson-Crowell		Korsmeyer-Peppas		
**1–8 h**		**R^2^**	***K*_0_**	**R^2^**	***K_H_***	**R^2^**	**k**	**R^2^**	**k**	**n_r_**
	H1*	0.89	9.58	0.97	11.88	0.9	−0.06	0.98	1.06	0.52
	H1.5*	0.84	9.31	0.94	10.81	0.85	−0.05	0.97	1.03	0.52
	H2*	0.82	10.64	0.92	15.36	0.84	−0.08	0.95	1.1	0.59
**24–144 h**	H1*	0.99	33.86	0.96	23.85	0.99	−0.003	0.93	1.26	0.22
	H1.5*	0.93	34.76	0.94	22.56	0.94	−0.004	0.93	1.22	0.25
	H2*	0.95	44.54	0.97	27.26	0.96	−0.008	0.97	1.3	0.27

R^2^: correlation coefficient; *K*: proportionality constant; n_r_: release exponent.
